# Conversion of Biowaste Asian Hard Clam (*Meretrix lusoria*) Shells into White-Emitting Phosphors for Use in Neutral White LEDs

**DOI:** 10.3390/ma9120979

**Published:** 2016-12-02

**Authors:** Tsung-Yuan Chang, Chih-Min Wang, Tai-Yuan Lin, Hsiu-Mei Lin

**Affiliations:** 1 Institute of Optoelectronic Sciences, National Taiwan Ocean University, 2 Pei Ning Road, Keelung 20224, Taiwan; 20088002@mail.ntou.edu.tw; 2 Department of Bioscience and Biotechnology, National Taiwan Ocean University, 2 Pei Ning Road, Keelung 20224, Taiwan; cmwang@ntou.edu.tw; 3 National Taiwan Ocean University Center of Excellence for the Oceans, 2 Pei Ning Road, Keelung 20224, Taiwan

**Keywords:** shell waste, solid-state reaction, carbothermic reaction, phosphor, near-UV

## Abstract

The increasing volume and complexity of waste associated with the modern economy poses a serious risk to ecosystems and human health. However, the remanufacturing and recycling of waste into usable products can lead to substantial resource savings. In the present study, clam shell waste was first transformed into pure and well-crystallized single-phase white light-emitting phosphor Ca_9_Gd(PO_4_)_7_:Eu^2+^,Mn^2+^ materials. The phosphor Ca_9_Gd(PO_4_)_7_:Eu^2+^,Mn^2+^ materials were synthesized by the solid-state reaction method and the carbothermic reduction process, and then characterized and analyzed by means of X-ray diffraction (XRD) and photoluminescence (PL) measurements. The structural and luminescent properties of the phosphors were investigated as well. The PL and quantum efficiency measurements showed that the luminescence properties of clam shell-based phosphors were comparable to that of the chemically derived phosphors. Moreover, white light-emitting diodes were fabricated through the integration of 380 nm chips and single-phase white light-emitting phosphors (Ca_0.979_Eu_0.006_Mn_0.015_)_9_Gd(PO_4_)_7_ into a single package of a white light emitting diode (WLED) emitting a neutral white light of 5298 K with color coordinates of (0.337, 0.344).

## 1. Introduction

Shellfish cultivation is an expanding economic activity worldwide. However, intensive shellfish production generates a large volume of waste consisting of shells. Recycling shell waste can be a good alternative to simply disposing of it, in terms of both ameliorating environmental problems and yielding economic benefits [1]. According to the Taiwan Fisheries Yearbook, about 50,000 tons of clam are generated in Taiwan annually, and the shell accounts for about 70% of the total weight of each clam. Clam shells are rich in calcium carbonate (CaCO_3_, approximately 95 wt %) in the form of aragonite or calcite, which gives them the potential to substitute for commercial fillers (e.g., precipitated CaCO_3_), for uses in plastics [2,3,4,5], and for uses as catalysts [6,7,8], and nanomaterials [9]. Given that these shells are cheap and abundant in nature, the capacity to convert them into phosphors could be of great benefit.

White light-emitting diodes (WLEDs) have attracted considerable attention because they promise high brightness, compact size, low weight, long lifetime, and a reduction in environmental pollution, and are thus expected to replace traditional fluorescent and incandescent lamps [10]. The first phosphor-converted WLED (pc-WLED) was fabricated by combining yellow-emitting phosphor (YAG:Ce^3+^) with a blue light-emitting diode (LED) (InGaN chip). Subsequently, pc-WLEDs were quickly adopted for commercial applications and have been used as a new generation light source for general illumination and display in various industries [11].

To solve the lack of a red component of the pc-WLED using the single YAG:Ce phosphor pumped by the blue LED chip, an alternative method has been employed that uses a UV LED chip coated with tricolor phosphors. However, the use of multiple emitting components for white LEDs can make the device very complicated. Moreover, the production price may be high and the control of color balance may also become difficult. Consequently, a mixture of single-phased phosphor with blue-to-red emission bands and a near-ultraviolet or ultraviolet chip system has been proposed [12]. White light emission can be generated by co-doping ions based on the energy transfer mechanism, which has been a hot area of research for WLEDs in recent years. Ca_9_Gd(PO_4_)_7_:Eu^2+^,Mn^2+^ (CGP:Eu^2+^,Mn^2+^) is a typical example of a single-phased white-emitting phosphor in which the light emission occurs as the result of energy transfer from Eu^2+^ to Mn^2+^ [12,13].

However, to the best of our knowledge, there have been no previous reports on the conversion of clam shell waste into single-phase white light-emitting phosphor or its potential benefits. In this work, we attempted to alter the structure of clam shell waste through heat treatment and use it as a calcium source to prepare the single-phase white light-emitting phosphor Ca_9_Gd(PO_4_)_7_:Eu^2+^,Mn^2+^. The phosphors were synthesized by the solid-state reaction method and the carbothermic reduction process, and then characterized and analyzed by means of X-ray diffraction (XRD) and photoluminescence (PL). The structural and the luminescent properties of Eu^2+^ and Mn^2+^ co-doped Ca_9_Gd(PO_4_)_7_ phosphors were investigated, and the energy transfer mechanism between Eu^2+^ and Mn^2+^ were also studied. Finally, we succeeded in fabricating white-emitting near-UV LEDs and thoroughly examined their optical properties.

## 2. Methods and Materials

### 2.1. Materials and Synthesis

First, biowaste clam shells (species: *Meretrix lusoria*) were thoroughly cleaned using distilled water and then dried in air. Next, the shells were heated at 500 °C for 2 h and then ground in an agate mortar to obtain clam shell powder. The conventional solid-state reaction was then employed to fabricate Ca_9_Gd_(0.994−*x*)_(PO_4_)_7_:0.006Eu^2+^,*x*Mn^2+^ phosphors. The starting materials used were (NH_4_)_2_HPO_4_, Gd_2_O_3_, Eu_2_O_3_, and MnCO_3_, with the clam shell powder serving as the CaCO_3_ source. The stoichiometric mixtures were homogeneously ground in an agate mortar and then heated at 1300 °C for 8 h in a covered crucible imbedded in active carbon. Then, the sample was cooled slowly to room temperature, and the white powder of Ca_9_Gd_(0.997−*x*)_(PO_4_)_7_:0.006Eu^2+^,*x*Mn^2+^ phosphors were obtained.

### 2.2. Measurements and Characterization

The crystal structures of the as-synthesized products were recorded with a Bruker D2 Phaser desktop XRD (Bruker Co., Karlsruhe, Germany) with Cu Kα (λ = 0.15406 nm) radiation. PL was investigated with a Horiba Jovin Yvon Fluoromax 4 equipped (Horiba Ltd., Edison, NJ, USA) with a Xe-arc lamp (Horiba Ltd.) at room temperature. The Commission Internationale de l’Éclairage’s (CIE) chromaticity coordinates, correlated color temperature (CCT) and colour rendering index (CRI), were measured by an LED Portable Lighting Measuring Equipment equipped with an integrating sphere (Rainbow Light Technology Co., Ltd., Taoyuan, Taiwan).

## 3. Results and Discussion

### 3.1. Phase Identification and Crystal Structure

#### 3.1.1. Initial Material

**Figure 1:**
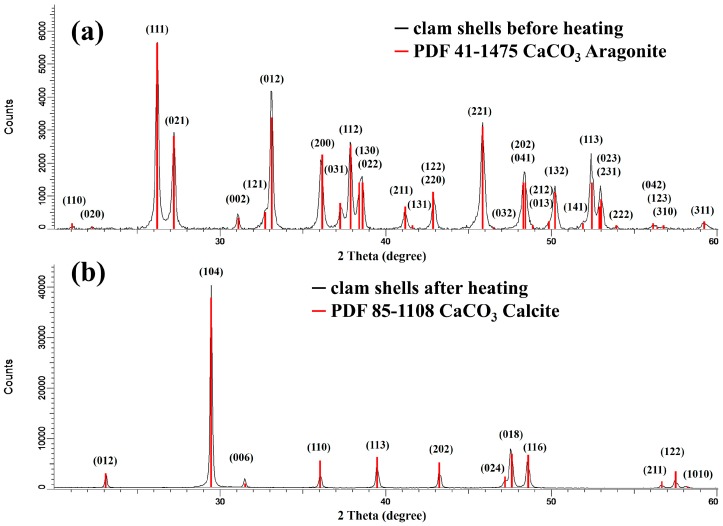
Powder X-ray diffraction (XRD) patterns of clam shells before heating (**a**); and clam shells after heating at 500 °C for 2 h (**b**).

Figure 1 shows the XRD patterns of the clam shells before heating (a) and after heating (b), and these patterns are consistent with the aragonite form (JCPDS 41-1475) and calcite form (JCPDS 85-1108), respectively. It was found that all the positions and relative intensities were in good agreement with the simulated patterns and no discernible impurity phase was detected. The XRD results thus indicate that the structure of the clam shells was successfully transformed from aragonite into calcite through the heat treatment, which removed the organic matter from the shells. The hardness of calcite (Mohs hardness = 3) is lower than that of aragonite (Mohs hardness = 3.5–4), such that when the transformed shells were used as a source of calcium carbonate, the subsequent milling treatment was far easier and more efficient. The colored pictures of the clam shells before and after cleaning and of the clam-based CaCO_3_ powder are shown in the Supplementary Materials Figure S1. The color of the clam-based CaCO_3_ powder was gray, which was attributed to carbon residue. The trace element of impurity in the clam-based CaCO_3_ powder was investigated by inductively coupled plasma-mass spectrometer (ICP-MS). The determination of Ba, Pb, Fe, Sr, Mg, K, Na, and Mn of the clam-based CaCO_3_ powder was performed by ICP-MS, and the data are shown in Table S1.

#### 3.1.2. Crystal Structure

**Figure 2:**
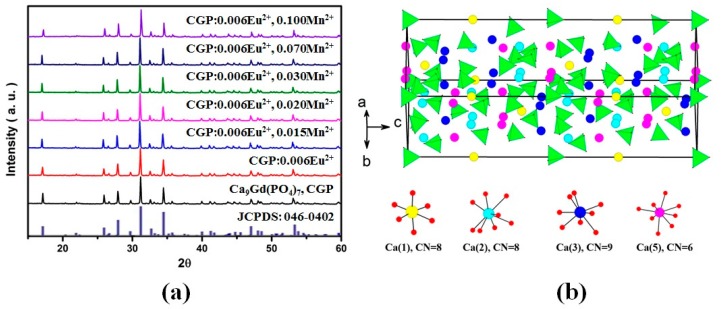
(**a**) XRD patterns of the representative CGP, CGP:0.006Eu^2+^,*x*Mn^2+^ (*x* = 0.015–0.1) phosphors, and standard pattern Ca_9_Y(PO_4_)_7_ (JCPDS 46-0402); (**b**) Representation of the Ca_9_Gd(PO_4_)_7_ supercell and four different coordinations of Ca^2+^ ions.

Figure 2a presents the XRD patterns of as-prepared CGP powders without doping rare earth ions, CGP:0.006Eu^2+^, CGP:0.006Eu^2+^,*x*Mn^2+^ (*x* = 0.015–0.1), and the standard data for Ca_9_Y(PO_4_)_7_ (JCPDS card No. 46-0402). The results show that all the diffraction peaks of the samples matched well with the Ca_9_Y(PO_4_)_7_ standard pattern (JCPDS 46-0402), which belongs to the rhombohedral phase with the space group R3c (No. 161). Representations of the unit cell, supercell, and coordination polyhedra for the cation sites in CGP are shown in Figure 2b. The Ca(1) and Ca(2) sites are 8-fold coordinated, the Ca(3) and Ca(5) sites are 9- and 6-fold coordinated, respectively, and the Ca(4), Ca(6) sites are vacant. Considering the ionic radii and charge balance, it was concluded that the Eu^2+^ and Mn^2+^ ions replace the Ca^2+^ sites in CGP. No impurity peaks were observed, indicating that both the Eu^2+^ and Mn^2+^ ions were completely dissolved into the Ca_9_Gd(PO_4_)_7_ host lattice. The morphologies of the clam-based phosphor are shown in Figure S2a. The phosphor grain size with an average size of 6 μm was characterized by regular crystallites as shown in the enlarged SEM image of Figure S2b.

### 3.2. Luminescence Properties

#### 3.2.1. Luminescent Spectra of Eu^2+^ and Mn^2+^ in the CGP

**Figure 3:**
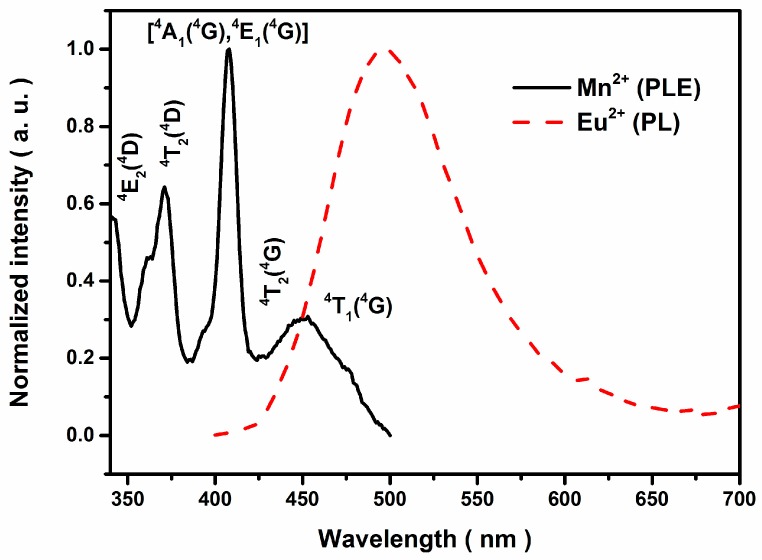
Photoluminescence (PL) and photoluminescence excitation (PLE) spectra of CGP:0.006Eu^2+^ (dash line) and CGP:0.02Mn^2+^ (solid line).

**Figure 4:**
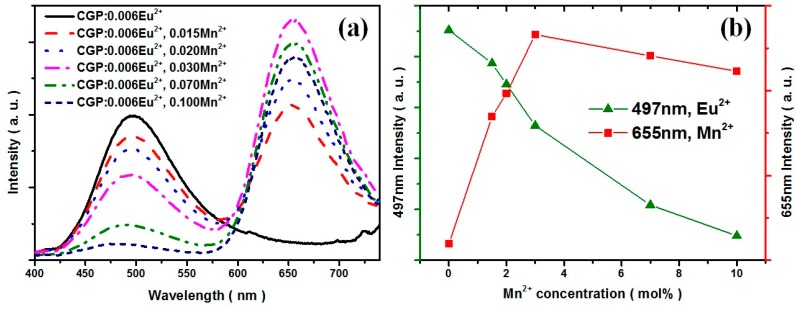
(**a**) The emission spectra of CGP:0.006Eu^2+^,*x*Mn^2+^ (*x* = 0–0.1) phosphors under the excitation of 380 nm; (**b**) The emission intensity of Eu^2+^ (monitored at 497 nm) and Mn^2+^ (monitored at 655 nm) versus Mn^2+^ concentrations.

The effective resonance energy transfer (ET) from Eu^2+^ to Mn^2+^ shown in Figure 3 was expected based on the observed significant spectral overlap between the emission band centered at 497 nm of CGP:Eu^2+^ (dash line) and the excitation band centered at 453 nm of CGP:Mn^2+^ (solid line). The PL spectrum showed an intense emission broad band centered at 497 nm, which was assigned to the 4f^6^5d^1^ → 4f^7^ transitions of CGP:Eu^2+^. The photoluminescence excitation (PLE) spectrum of CGP:Mn^2+^ contained several bands centered at 341, 371, 408, 418, and 453 nm, corresponding to the transitions from the ^6^A_1_ (^6^S) to ^4^E (^4^D), ^4^T_2_ (^4^D), [^4^A_1_ (^4^G), ^4^E (^4^G)], ^4^T_2_ (^4^G), and ^4^T_1_ (^4^G) levels, respectively [12,13]. Therefore, a resonance type ET_Eu→Mn_ was expected. Additionally, the low intensity humps at ~611 nm and ~673 nm of CGP:0.006Eu^2+^ phosphor are assigned to the ^5^D_0_ → ^7^F*_j_* (*j* = 2 and 3) transition of Eu^3+^ [14], which is considered to originate from the remaining Eu^3+^. A similar result was also observed in Figure 4a, which indicated that Eu^3+^ residue existed in CGP:0.006Eu^2+^,*x*Mn^2+^ phosphors.

Figure 4a shows the PL spectra of CGP:0.006Eu^2+^,*x*Mn^2+^ phosphors with different doping concentrations, *x*, which was measured under the excitation wavelength of 380 nm. With increasing Mn^2+^ doping content, the emission intensity of the Mn^2+^ ions was also increased and reached a maximum when *x* was equal to 0.07, whereas the intensity of the Eu^2+^ ions was found to decrease remarkably from *x* = 0.015 to 0.10. These results indicate that the energy was transferred from the Eu^2+^ to Mn^2+^ ions. The emission intensity of the Mn^2+^ ions reached a maximum at *x* = 0.03 and then began to decrease as a result of the concentration quenching of the Mn^2+^ ions. With respect to the mechanism of energy transfer in phosphors, Blasse [15,16] has pointed out that the critical transfer distance (*R*_c_) is approximately equal to twice the radius of a sphere with the equation:

(1) $$R_{c}=2{\left[\frac{3V}{4\pi X_{c}N}\right]}^{\frac{1}{3}}$$

where *N* is the number of molecules in the unit cell and *V* is the unit cell volume. If the critical concentration *X*_c_ is used in the above equation, *R*_c_ can be obtained. The critical concentration *X*_c_ is defined as the concentration at which the luminescence intensity of Eu^2+^ reduces to half of that for the sample in the absence of Mn^2+^. Figure 4b indicates that when the Mn^2+^ content was 4.2%, the Eu^2+^ intensity was decreased to approximately half. Accordingly, *X*_c_ is about 0.048, *N* = 54, and *V* = 3536.6 Å^3^. The critical distance *R*_c_ was thus estimated to be about 13.8 Å.

#### 3.2.2. Energy Transfer Mechanism of CGP:Eu^2+^,Mn^2+^ Phosphors

**Figure 5:**
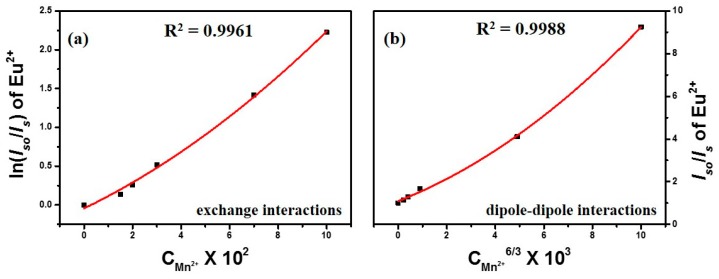

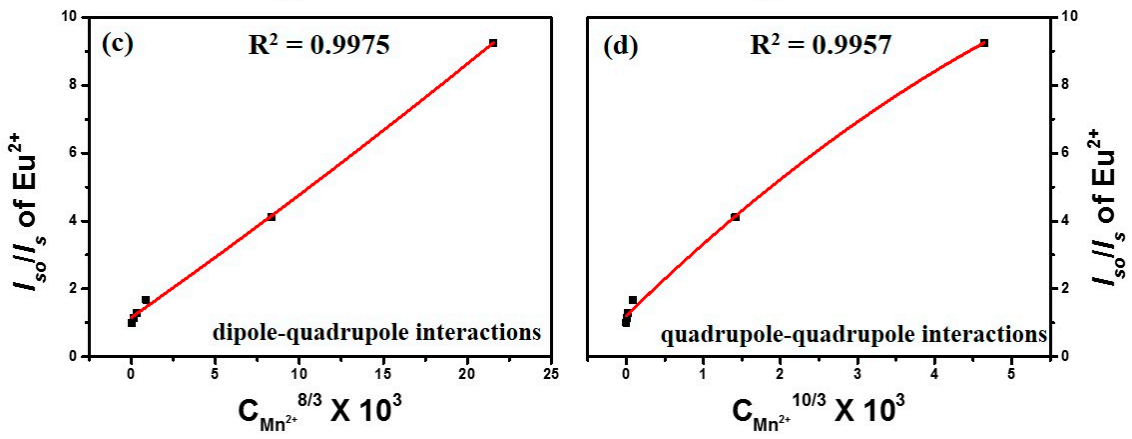
(**a**) Dependence of ln(*I*_S0_/*I*_S_) of Eu^2+^ on *C*_Mn^2+^_; and of *I*_S0_/*I*_S_ of Eu^2+^ on (**b**) *C*_Mn^2+^_^6/3^; (**c**) *C*_Mn^2+^_^8/3^; (**d**) *C*_Mn^2+^_^10/3^.

From the Equation (1), the critical distance *R*_c_ was calculated to be about 13.8 Å. This value is larger than 5 Å, indicating little possibility of energy transfer via the exchange interaction mechanism [17]. Thus, the electric multipolar interaction can take place for energy transfer between the Eu^2+^ and Mn^2+^ ions. According to Dexter’s energy transfer formula for exchange and multipolar interactions [18,19], the following relationship can be obtained:

(2) $$\ln\left(\frac{I_{S0}}{I_{S}}\right)\;\propto \;C$$

(3) $$\frac{I_{S0}}{I_{S}}\;\propto \;C^{\frac{\alpha }{3}}$$

where *C* is the concentration of Mn^2+^ and *I*_S0_ and *I*_S_ are the luminescence intensities of the sensitizer (Eu^2+^) without and with the activator (Mn^2+^) present, respectively. ln(*I*_S0_/*I*_S_) ∝ *C* corresponds to the exchange interaction and (*I*_S0_/*I*_S_) ∝ *C*^α/3^ with α = 6, 8, and 10 corresponding to dipole-dipole, dipole-quadrupole, and quadrupole-quadrupole interactions, respectively. The relationships of ln(*I*_S0_/*I*_S_) ∝ C and (*I*_S0_/*I*_S_) ∝ *C*^α/3^ are illustrated in Figure 5, which indicates that a linear behavior was observed only when α = 8, implying that the energy transfer from Eu^2+^ to Mn^2+^ occurred via a dipole-quadrupole mechanism, a finding which is consistent with that of previous investigations [12,13]. Therefore, the electric dipole-quadrupole interaction predominates in the energy-transfer mechanism from the Eu^2+^ to Mn^2+^ ions in clam-based CGP. Considering the dipole-quadrupole interaction, the critical distance from a sensitizer to an acceptor can be estimated by the spectral overlap method. Hence, *R*_c_ can be obtained from the Equation (4) as in [17]

(4) $$R_{c}{}^{8}=3.024\times 10^{12}\lambda_{S}^{2}\;f_{q}\int \frac{F_{S}\left(E\right)F_{A}\left(E\right)dE}{E^{4}}$$

where *f*_q_ is the oscillator strength of the involved absorption transition of the acceptor (Mn^2+^), λ_S_ (in angstroms) is the wavelength position of the sensitizer’s emission, *E* is the energy involved in the transfer (in electronvolts), and ∫*F*_S_(*E*)·*F_A_*(*E*)·d*E*/*E*^4^ represents the spectral overlap between the normalized shapes of the Eu^2+^ emission *F*_S_(E) and the Mn^2+^ excitation *F*_A_(*E*). The spectral overlap is calculated to be about 0.03448 eV^−5^. Using the above equation with *f*_q_ = 10^−10^, the critical distance *R*_c_ was estimated to be 11.3 Å, which agrees, approximately, with that obtained by using the concentration-quenching method. This result further reveals that the mechanism of energy transfer from the Eu^2+^ to Mn^2+^ ions is mainly due to a dipole-quadrupole interaction.

#### 3.2.3. CIE Coordinates of CGP:Eu^2+^,Mn^2+^

**Figure 6:**
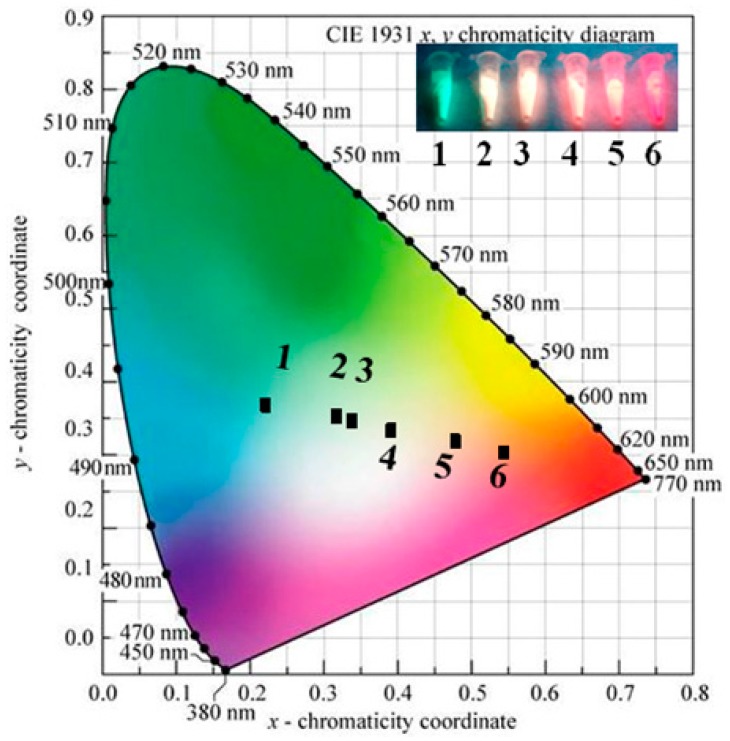
Commission Internationale de l’Éclairage’s (CIE) chromaticity diagram of CGP:0.006Eu^2+^,*x*Mn^2+^ phosphors under 380 nm excitation: (1) *x* = 0; (2) *x* = 0.015; (3) *x* = 0.02; (4) *x* = 0.03; (5) *x* = 0.07; (6) *x* = 0.1. The insets show CGP:0.006Eu^2+^,*x*Mn^2+^ phosphors irradiated under 365 nm UV light in a lamp box.

Figure 6 shows the Commission Internationale de l’Éclairage’s (CIE) 1931 chromaticity diagram of a single phased emission-tunable phosphor CGP:0.006Eu^2+^,*x*Mn^2+^ under 380 nm excitation. The chromaticity coordinates (*x*, *y*) were measured as (0.220, 0.370), (0.316, 0.355), (0.337, 0.348), (0.390, 0.335), (0.477, 0.320), and eventually (0.543, 0.305) for CGP:0.006Eu^2+^,*x*Mn^2+^ phosphors with *x* = 0, 0.015, 0.02, 0.03, 0.07, and 0.1, respectively. These results indicate that changing the Mn^2+^ concentration can tune the color hue from blue-green (solely 0.006Eu^2+^, point 1) through white light (0.006Eu^2+^/0.15Mn^2+^, point 2; 0.006Eu^2+^/0.02Mn^2+^, point 3) and eventually to red (0.006Eu^2+^/0.1Mn^2+^, point 6) in the visible spectral region. The insets of Figure 6 show photographs of CGP:0.006Eu^2+^,*x*Mn^2+^ phosphors with different Mn^2+^ contents in a 365 nm UV lamp box.

#### 3.2.4. Quantum Efficiencies of Clam-Based and Chem-Based CGP:Eu^2+^,Mn^2+^ Phosphors

The PL spectra of the obtained phosphor and the phosphor obtained by high purity materials were studied. The CGP:0.006Eu^2+^,0.015Mn^2+^ phosphors for which reactants CaCO_3_ were prepared from high purity reagent or clam shells were named chem-based and clam-based CGP:0.006Eu^2+^,0.015Mn^2+^ materials, respectively. The PL intensities of these two materials were shown in the Figure S3. While in the blue-green region, their intensities were comparable; the red emission of clam-based CGP:0.006Eu^2+^,0.015Mn^2+^ was stronger than that od the chem-based one. Additionally, the quantum efficiency measurements of clam-based CGP:Eu^2+^,Mn^2+^ phosphor and chem-based CGP:Eu^2+^,Mn^2+^ phosphor were performed. The quantum efficiencies with error bar (error bar is the standard deviation, *n* = 3) of clam-based and chem-based CGP:0.006Eu^2+^, 0.015Mn^2+^ phosphors were 11.9% ± 0.2% and 11.2% ± 0.2%, respectively (Figure S4). The quantum efficiency of clam-based phosphor was comparable to that of chem-based phosphor.

#### 3.2.5. Thermal Stability of Clam-Based and Chem-Based CGP:Eu^2+^,Mn^2+^ Phosphors

Moreover, the thermal quenching tests of phosphors to confirm that the thermal stability of shell source (clam-based) CGP:Eu^2+^,Mn^2+^ was comparable to high purity (chem-based) CGP:Eu^2+^,Mn^2+^ materials are shown in Figure S5. Figure S5 represents the temperature-dependent PL spectra of CGP:0.006Eu^2+^, 0.015Mn^2+^ (a, chem) and (b, clam) excited at 385 nm from 25 to 200 °C; both PL intensities slowly decreased with increasing temperature. It can be seen that the chem-based CGP:0.006Eu^2+^,0.015Mn^2+^ phosphors have an obvious decreasing trend with increasing temperature. The luminescence intensities at 497 nm and 654 nm dropped to 50% of the initial intensity when the temperatures (*T*_50_) were 75 and 130 °C, respectively (Figure S5c). For the clam-based CGP:0.006Eu^2+^,0.015Mn^2+^ phosphors, the T_50_ of 497 nm and 652 nm were 83 and 137 °C, respectively (Figure S5d). Thus, it can be seen that the CGP:0.006Eu^2+^,0.015Mn^2+^ (clam) phosphor exhibits slightly higher thermal stability than CGP:0.006Eu^2+^,0.015Mn^2+^ (chem) phosphor. Thus, the clam-based phosphor is preferable with respect to its thermal quenching properties than chem-based phosphor.

#### 3.2.6. White-Emitting LED Packages by Near-UV Chip

The spectrum of a phosphor-converted (pc)-LED lamp fabricated with a 380 nm near-UV LED chip and a white-emitting clam-based CGP:0.006Eu^2+^,0.015Mn^2+^ driven by a 350 mA current is shown in Figure S6a. Accordingly, the correlated color temperature, CRI, and CIE color coordinates were determined to be 5298 K, 55.2, and (0.337, 0.344), respectively, as shown in Figure S6b. Figure S6c shows the appearance of the phosphor-converted white LED lamp, and Figure S6d shows the white light emission from the LED driven by a 350 mA current.

### 3.3. The Production Cost of Clam-Based Phosphors

The production cost of clam-based CaCO_3_ have been estimated, and the synthesis cost of clam-based CaCO_3_ was about 0.48 USD per kg described in the supporting information. Note that the synthesis cost could decrease if clam-based CaCO_3_ were massively produced. On the other hand, the cost of commercial CaCO_3_ (XR-LCAL003-25KG, Uni-Onward Co., Taipei, Taiwan) per kg is about 3.6 USD according to the invoice of local agents. Thus, it can be found that the cost of clam-based CaCO_3_ could be lower than that of commercial CaCO_3_. Moreover, we expect the development of an all calcium-based host for phosphors, such as Gd-free Ca_3_(PO_4_)_2_ compound, which will be cost effective for the phosphor applications in our future work.

## 4. Conclusions

To summarize, in this study, biowaste clam shells were converted into single-phase white light-emitting phosphor Ca_9_Gd(PO_4_)_7_:Eu^2+^,Mn^2+^ materials via the solid-state reaction method and the carbothermic reduction process; the structural and luminescent properties of the phosphors were comparable to the chemically derived phosphors. Spectroscopic data analysis indicated that the energy transfer from the Eu^2+^ ions to the Mn^2+^ ions took place through a dipole-quadrupole interaction and that the critical distance was 13.8 Å. The emission color can be easily modulated from blue-green through white light and eventually to red by adjusting the Mn^2+^ content. Moreover, a white light LED was also fabricated through the integration of a 380 nm near-UV chip and a single-phased white light phosphor (CGP:0.006Eu^2+^,0.015Mn^2+^), and the LED emitted a neutral white light with a color temperature of 5298 K and color coordinates of (0.337, 0.344). These results show that converting biowaste clam shells into phosphor Ca_9_Gd(PO_4_)_7_:Eu^2+^,Mn^2+^ materials may be an effective means of providing single-phase white light phosphor applications. Furthermore, the results of our study should also provide motivations for further research using various types of biogenic waste to produce low-cost and ecologically friendly optoelectronic devices.

## Supplementary Materials

The following are available online at www.mdpi.com/1996-1944/9/12/979/s1.
