# Supplementary material for: Conversion of Biowaste Asian Hard Clam (Meretrix lusoria) Shells into White-Emitting Phosphors for Use in Neutral White LEDs
Source: Materials (Basel). 2016 Dec 2;9(12):979. doi: 10.3390/ma9120979 (PMC5457013; doi:10.3390/ma9120979)
Supplement: Supplementary file 1 [file materials-09-00979-s001.pdf]

Supplementary Materials: Conversion of Biowaste Asian Hard Clam (*Meretrix lusoria*) Shells into White-Emitting Phosphors for Use in Neutral White LEDs

Tsung-Yuan Chang, Chih-Min Wang, Tai-Yuan Lin and Hsiu-Mei Lin

1. Observations and Measurements

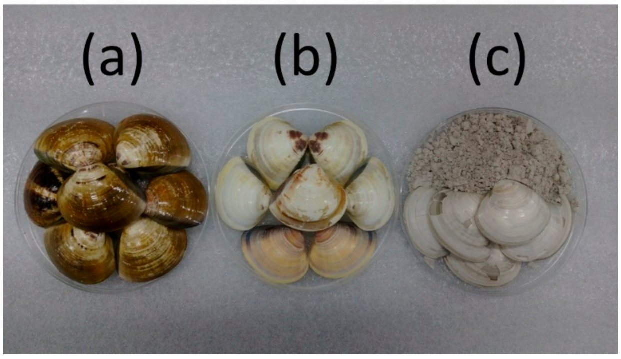

Figure S1. Color picture of the clam shells before (**a**); and after (**b**) cleaning; and the clam-based $CaCO_3$ power with unmilled shells (**c**).

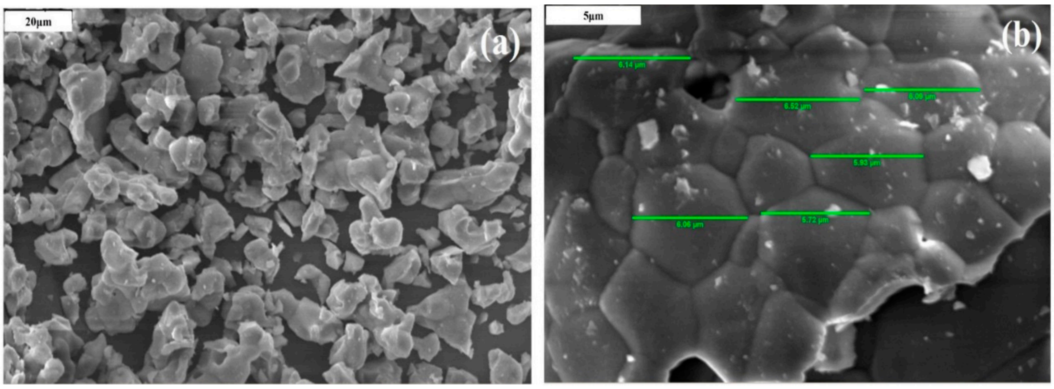

Figure S2. (**a**) Scanning electron microscopy (SEM) image of the morphologies of the clam-based phosphor; and (**b**) enlarged SEM image with regular crystallites.

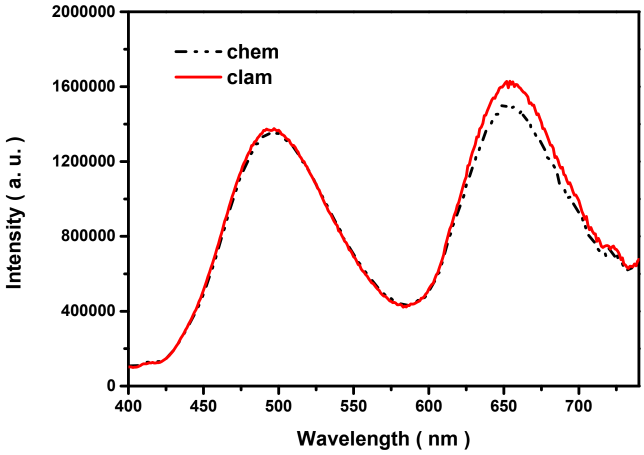

Figure S3. The emission spectra of chemical-based (**black**, dot) and clam-base (**red**, line) CGP: $0.006Eu^{2+}$,$0.015Mn^{2+}$ under the excitation of 380 nm.

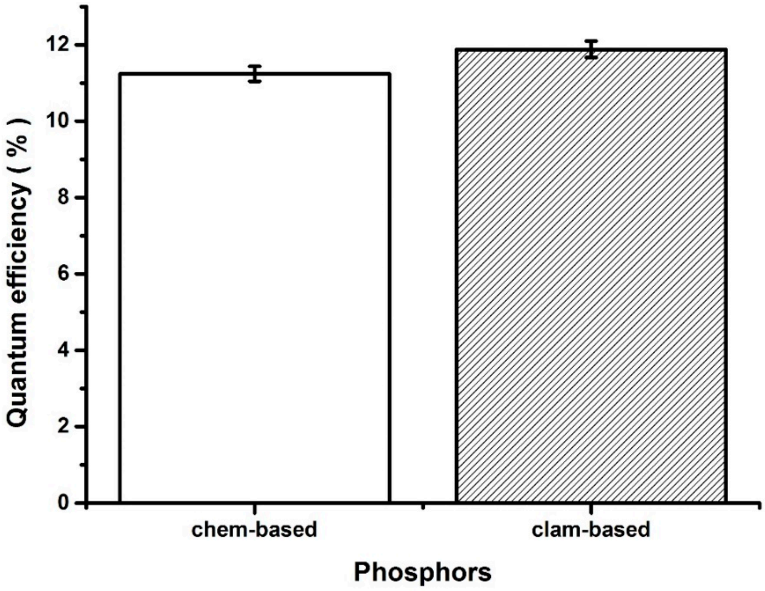

Figure S4. The quantum efficiencies of chem-based (**empty**); and clam-based (**fill**) CGP:0.006Eu^{2+},0.015Mn^{2+} phosphors.

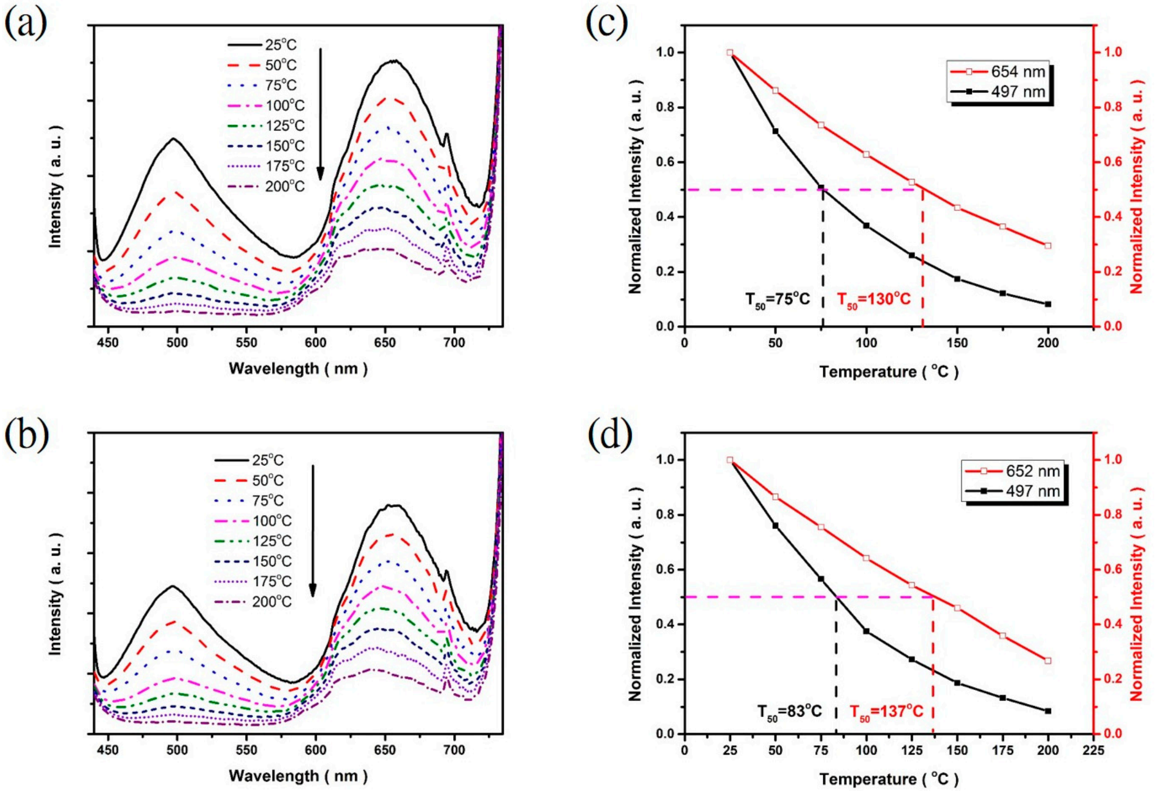

Figure S5. (**a**) Temperature-dependence PL spectra of CGP:0.006Eu^{2+},0.015Mn^{2+} (chem) under 385 nm excitation; (**b**) Thermal quenching data of CGP:0.006Eu^{2+},0.015Mn^{2+} (clam) excited at 385 nm; (**c**,**d**) Normalized PL intensities as a function of temperature.

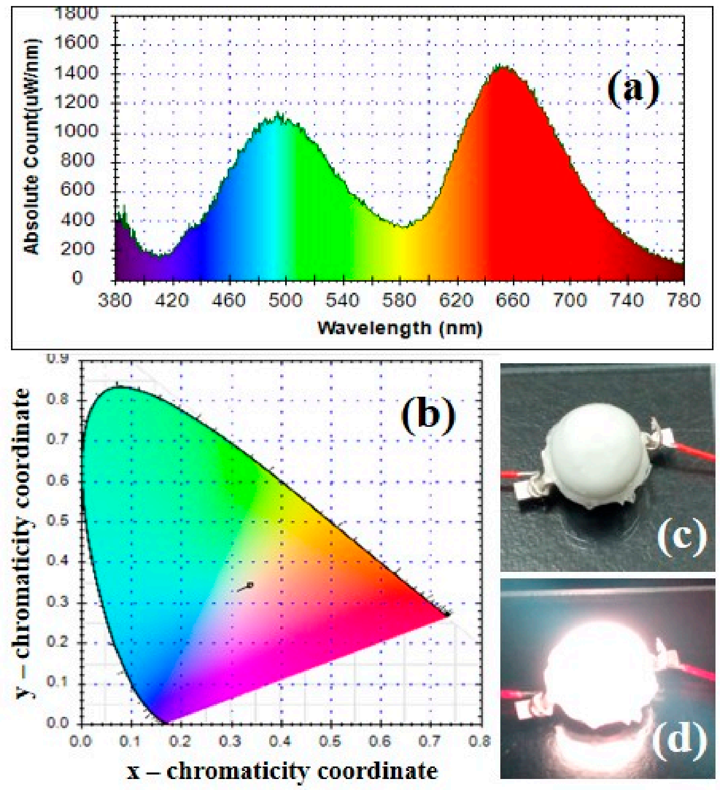

Figure S6. (**a**) The spectra; and (**b**) CIE chromaticity coordinates of white LED lamps fabricated using a near-UV 380 nm chip combined with a white-emitting clam-based phosphor CGP:0.006Eu^{2+},0.015Mn^{2+} driven by a 350 mA current; The inset (**c**,**d**) shows a photograph of the LED lamp package.

Table S1. Results of ICP-MS elemental analysis for the clam-based $CaCO_3$.

	**A.C.S. Reagent $CaCO_3$ (%) ***	**Clam-Based $CaCO_3$ (%)**
Ba	≤0.01	0.005 ± 0.002
Pb	≤0.0010	0.000029 ± 0.000004
Fe	≤0.003	0.0199 ± 0.002
Mg	≤0.02	0.0165 ± 0.001
K	≤0.01	0.0065 ± 0.002
Na	≤0.1	0.57 ± 0.04
Sr	≤0.1	0.153 ± 0.003
Mn	-	0.0014 ± 0.00007

* J. T. BAKER, Product Number: 1288-01.

2. Estimation of the Production Cost of Clam-Based $CaCO_3$

For example: processing cost for 3 kg of clam shells (raw material) (The production yield of purified $CaCO_3$ is approximate 95 wt %).

(1) The cleaning cost is negligible.
- Taiwian Water Corporation
  - 0.31 USD for 1000 L water.
  - Clean process consumed about 10 L, cost 0.0031 USD (negligible)

(2) The electricity cost is 1.39 USD
- Taiwian Power Corporation
  - 0.12 USD for 1000 W·h
  - Box Furnaces (Linberg BF51794C, 3500 W), 18.4 L free space, using 60% space, 500 °C for 2.5 h (full power) consumed 8750 W, cost 1.08 USD.
  - Grinder (1200 W), full power for 2 h consumed 2400 W, cost 0.30 USD.

From (1) and (2), it is found that since the addition water cost is negligible and power cost is about 1.38 USD, therefore, the total cost is ~1.38 USD for 2.85 kg (3 kg × 95 wt %) clam-based $CaCO_3$.

Accordingly, is ~0.48 USD per kg for clam-based $CaCO_3$.

3. Estimation of the Production Cost of CGP:0.006Eu^{2+},0.015Mn^{2+} Phosphor

For example: processing cost for 1 kg CGP:0.006Eu^{2+},0.015Mn^{2+} phosphor

(1) Reactant cost is 428.11 (commercial $CaCO_3$) and 425.81 (clam-based $CaCO_3$) USD
Reactant:

I $CaCO_3$ (Please refer to supporting information):

a commercial (XR-LCAL003-25KG): 754.237 g × 0.0036 (USD/g) = 2.7 USD
b clam-based: 754.237 g × 0.00048 (USD/g) = 0.4 USD

II Gd_2O_3(AF-11291-100G): 155.013 g × 49.4 (NTD/g) = 7658 NTD = 232 USD
III $(NH_4)_2HPO_4$(VE-V900033-500G): 806.74 g × 2 (NTD/g) = 1614 NTD = 48.9 USD
IV Eu_2O_3(AF-11300-25G): 8.125 g × 585.2 (NTD/g) = 4754.75 NTD = 143.1 USD
V $MnCO_3$(AF-14324-500G): 13.272 g × 3.5 (NTD/g) = 46.45 NTD = 1.41 USD

(2) The electricity cost is 5.34 USD
- Taiwian Power Corporation
  - 0.12 USD for 1000 W·h
  - Box Furnaces (3500 W), 18.4 L free space, using 60% space, 1300 °C for 12 h (full power) consumed 42,000 W, cost 5.04 USD.
  - Grinder (1200 W), full power for 2 h consumed 2400 W, cost 0.30 USD.

From (1) and (2), it is found that the total cost is ~433.45 USD (commercial $CaCO_3$) and 431.15 (clam-based $CaCO_3$) for 1 kg CGP:0.006Eu^{2+},0.015Mn^{2+} phosphor.

In summary, the $CaCO_3$ cost is small compared to the cost of the rare-earth precursors.
